# Responses of Photosystem I Compared with Photosystem II to Fluctuating Light in the Shade-Establishing Tropical Tree Species *Psychotria henryi*

**DOI:** 10.3389/fpls.2016.01549

**Published:** 2016-10-17

**Authors:** Wei Huang, Ying-Jie Yang, Hong Hu, Shi-Bao Zhang

**Affiliations:** ^1^Key Laboratory of Tropical Forest Ecology, Xishuangbanna Tropical Botanical Garden, Chinese Academy of SciencesYunnan, China; ^2^Key Laboratory of Economic Plants and Biotechnology, Kunming Institute of Botany, Chinese Academy of SciencesKunming, China

**Keywords:** constant high light, fluctuating light, PSI photoinhibition, excess excitation energy, shade-establishing species

## Abstract

Shade-establishing plants growing in the forest understory are exposed to constant high light or fluctuating light when gaps are created by fallen trees. Our previous studies indicate that photosystem I (PSI) is sensitive to constant high light in shade-establishing tree species, however, the effects of fluctuating light on PSI and photosystem II (PSII) in shade-establishing species are little known. In the present study, we examined the responses of PSI and PSII to fluctuating light in comparison to constant high light in the shade-establishing species *Psychotria henryi*. Accompanying with significant activation of cyclic electron flow (CEF), the P700 oxidation ratio was maintained at high levels when exposed to strong light either under fluctuating light or constant high light. Under moderate fluctuating light, PSI and PSII activities were remained stable in *P. henryi*. Interestingly, PSI was insusceptible to fluctuating light but sensitive to constant high light in *P. henryi*. Furthermore, both PSI and PSII were more sensitive to constant high light than fluctuating light. These results suggest that CEF is essential for photoprotection of PSI under fluctuating light in *P. henryi*. Furthermore, photoinhibition of PSI under high light in *P. henryi* is more related to the accumulation of reactive oxygen species rather than to P700 redox state, which is different from the mechanisms of PSI photoinhibition in *Arabidopsis thaliana* and rice. Taking together, PSI is a key determiner of photosynthetic responses to fluctuating light and constant high light in the shade-establishing species *P. henryi*.

## Introduction

Naturally, plants experience highly variable light conditions. Plants growing in forest understory experience more frequent, short-term light fluctuations due to leaves and stems of other plants above them in addition to clouds. In the understory of tropical rain forests, leaves of shade-establishing plants usually grow in a light environment of deep shade (<5% of full sunlight). When canopy gaps are created by fallen trees, leaves of shade-establishing plants may be exposed to fluctuating light or constant high light for several hours in a day. Under constant high light, excess light energy usually causes selective photodamage to photosystem II (PSII) (Barber and Andersson, 1992; Aro et al., 1993; Kitao et al., 2000; Barth et al., 2001). Generally, wild-type plants are capable of protecting photosystem I (PSI) when exposed to constant high light at normal growth temperature (Barth et al., 2001; Munekage et al., 2002, 2004; Suorsa et al., 2012; Kono et al., 2014; Tikkanen et al., 2014; Yamori et al., 2016). Interestingly, our previous studies indicated that both PSI and PSII are susceptible to constant high light in the shade-establishing tropical tree species *Psychotria rubra* (Huang et al., 2015b, 2016d). However, the responses of PSI and PSII to fluctuating light in shade-establishing plants are little known.

Previous studies have reported that at chilling-light stress photoinhibition of PSI occurs in the chilling-sensitive plants cucumber (Havaux and Davaud, 1994; Terashima et al., 1994; Sonoike, 1995; Kudoh and Sonoike, 2002), tobacco (Barth and Krause, 1999; Huang et al., 2016c) and *Arabidopsis thaliana* (Zhang and Scheller, 2004). At chilling temperature, the photoinhibition of PSI in cucumber occurred mainly due to the oxidative damage to PSI by hydroxyl radicals that are generated by a reaction between reduced iron–sulfur centers and hydroxyl peroxide (Sonoike, 1995, 2011). At normal growth temperature, PSI is insusceptible to constant high light in *A. thaliana* mainly due to significant activation of cyclic electron flow (CEF) (Munekage et al., 2002, 2004). In the CEF mutant *pgr5*-plants of *A. thaliana*, high light causes a large decrease in PSI activity (Munekage et al., 2002; Suorsa et al., 2012; Tikkanen et al., 2014). Previous studies suggested that over-reduction of PSI acceptor side was the main mechanism for PSI photoinhibiton in the *pgr5*-plants when exposed to high light (Munekage et al., 2002, 2004). Recently, a lot of studies suggested that photoinhibition of PSI in the *pgr5*-plants was mainly caused by the uncontrolled electron transport from PSII to PSI (Suorsa et al., 2012; Tikkanen et al., 2014, 2015; Chaux et al., 2015; Suorsa, 2015; Yamori and Shikanai, 2016; Yamori et al., 2016), which not only accelerates production of reactive oxygen species (ROS) at the acceptor side of PSI, but also induces over-reduction of PSI reaction centers. By comparison, in the shade-establishing tree species *P. rubra*, PSI photoinhibition is more related to electron flow from PSII to PSI rather than to PSI redox state (Huang et al., 2016d). These results suggested that the mechanisms of PSI photoinhibition may be different between *A. thaliana* and shade-establishing species. Consequently, it is assumed that the response of PSI to fluctuating light likely differs between shade-establishing species and the model plants *A. thaliana*.

A recent study reported that PSI photoinhibition under fluctuating light could occur even in the wild-type of rice (Yamori et al., 2016), which is consistent with the small decrease in PSI activity under fluctuating light for wild-type of *A. thaliana* (Kono et al., 2014). What is more, PSI is more sensitive to fluctuating light than constant high light in both wild-type and *pgr5*-plants of *A. thaliana* and rice (Kono et al., 2014; Yamori et al., 2016). For plants of shade-establishing tree species grown in forest understory, leaves are usually exposed to fluctuating light. If PSI activity is more sensitive to fluctuating light than constant high light, shade-establishing tree species are unable to optimize photosynthesis and plant growth under naturally fluctuating light conditions. Furthermore, the extent of PSI photoinhibition in the shade-establishing species *P. rubra* can be significantly affected by excess electron flow to PSI (Huang et al., 2016d). The production of ROS under fluctuating light is less than under constant high light due to low-light phases. As a result, we speculate that PSI is insusceptible to fluctuating light in shade-establishing tree species.

In addition to PSI, PSII is very susceptible to constant high light in shade-establishing tree species. After treatment with 2000 μmol photons m^-2^ s^-1^ at 25°C for 2 h, the maximum quantum yield of PSII, as indicated by *F*_v_/*F*_m_, decreased by 80% in detached leaves of *P. rubra* (Huang et al., 2016d). Severe PSII photoinhibition can depress electron flow from PSII (Tikkanen et al., 2014; Huang et al., 2015a,b), and thus the rate of CO_2_ assimilation and plant growth. Furthermore, the fast recovery of PSII activity is dependent on linear electron flow sustained by residual PSII activity for a number of reasons including provision of ATP, provision of reducing equivalents for the translational regulation of D1 protein, efficient translation of D1 protein, and integration of D1 protein into PSII reaction centers (Sun et al., 2006; Huang et al., 2010b). In order to optimize photosynthesis under fluctuating light in an understory environment, shade-establishing species should prevent severe PSII photoinhibition under fluctuating light. Thus, we speculate that PSII activity is less sensitive to fluctuating light than constant high light in shade-establishing species.

Since shade-establishing tree species typically experience fluctuating light conditions, understanding of the photosynthetic performances under fluctuating light is of help to forest succession. However, the photosynthetic responses to fluctuating light for leaves of shade-establishing tree species remain to be clarified. In the present study, we examined the responses of PSI and PSII to fluctuating light and constant high light in *P. henryi*, a shade-establishing tropical tree species. Interestingly, PSI and PSII in *P. henryi* are hardly damaged by treatment with fluctuating light alternating between 100 and 1294 μmol photons m^-2^ s^-1^ every 2 min for 60 min. Furthermore, PSI is less sensitive to fluctuating light than constant high light in *P. henryi*, which is opposite to *A. thaliana* and rice. Our results strongly suggested that the response of PSI to fluctuating light may be different between shade-establishing and sun-establishing plants owing to different mechanisms of PSI photoinhibition.

## Materials and Methods

### Plant Materials

The tropical tree species *P. henryi* Levl. (Rubiaceae) was chosen for study. *P. henryi* is a shade-establishing shrub species of rain forests native to south of China, and the maximum height of its plants is about 2 m. In our present study, plants of *P. henryi* grown naturally in forest understory with deep shade (light intensity < 5% of sunlight) in tropical rain forest in Xishuangbanna tropical botanical garden (21°54′ N, 101°46′ E) were used for photosynthetic measurements. After cutting off from plants, the branches were immediately immersed in water and brought back to our lab in 5 min, and the photosynthetic measurements were conducted subsequently.

### PSI and PSII Measurements

In our present study, the PSI and PSII parameters were measured simultaneously by Dual-PAM-100 (Heinz Walz GmbH, Effeltrich, Germany). After dark adaptation for 30 min, the minimum and maximum fluorescence and the maximum photo-oxidizable P700 were measured. The maximum quantum yield of PSII, *F*_v_/*F*_m_ = (*F*_m_–*F*_o_)/*F*_m_, was measured to monitor PSII activity. Other fluorescence parameters were calculated as follows (Genty et al., 1989; Hendrickson et al., 2004; Kramer et al., 2004): *Y*(II) = ($F_{m}^{\prime }$- F_s_)/$F_{m}^{\prime }$, NPQ = (*F_m_–*$F_{m}^{\prime }$)/$F_{m}^{\prime }$, qL = ($F_{m}^{\prime }$- F_s_)/($F_{m}^{\prime }$- $F_{o}^{\prime }$) × ($F_{o}^{\prime }$/*F*_s_). *F*_m_ is the minimum and maximum fluorescence measured after 30 min dark adaptation. $F_{o}^{\prime }$ and $F_{m}^{\prime }$ are the minimum and maximum fluorescence after light adaptation. *F*_s_ is the steady-state fluorescence after light adaptation. *F*_m_ and $F_{m}^{\prime }$ were measured upon illumination of a saturating pulse (300 ms and 10000 μmol photons m^-2^ s^-1^). *Y*(II) represents the effective quantum yield of PSII, NPQ indicates the non-photochemical quenching. qL indicates the fraction of PSII centers in the open state (with oxidized plastoquinone).

The PSI photosynthetic parameters were evaluated by Dual PAM-100, based on P700 oxidation signal (i.e., the difference in intensities of 830 and 875 nm pulse-modulated measuring light reaching the photodetector) (Klüghammer and Schreiber, 2008). The P700^+^ signals (*P*) may vary between minimal (P700 is fully reduced) and maximal levels (P700 is fully oxidized). The maximum level (*P*_m_) was determined with application of a saturation pulse (300 ms and 10000 μmol photons m^-2^ s^-1^) after pre-illumination with far-red light (Klüghammer and Schreiber, 1994, 2008). $P_{m}^{\prime }$ was determined similar to *P*_m_ but with actinic light instead of far-red light. *P*_m_ was recorded to estimate the maximum photo-oxidizable P700 (Huang et al., 2010a,b, 2013; Suorsa et al., 2012; Tikkanen et al., 2014; Yamori et al., 2016). In our present study, *P*_m_ was measured after 30 min dark adaptation. The quantum yield of PSI was calculated as *Y*(I) = ($P_{m}^{\prime }$–*P*)/*P*_m_, and the P700 oxidation ratio in a given actinic light was calculated as *Y*(ND) = *P*/*P*_m_. The fraction of P700 that cannot be oxidized by SP to the overall P700 was calculated as *Y*(NA) = (*P*_m_–$P_{m}^{\prime }$)/*P*_m_.

The electron transport rate (ETR) was calculated as ETRI = PPFD × 0.5 × abs I ×*Y*(I), and ETRII = PPFD × 0.5 × abs I ×*Y*(II), where 0.5 is the fraction of absorbed light reaching PSI or PSII, and abs I is absorbed irradiance taken as 0.84 of incident irradiance.

### Photoinhibitory Treatments

In the present study, light from a 635 nm light-emitting diode (LED) equipped in Dual-PAM-100 was used as actinic light for photoinhibitory treatments. In order to examine the response of PSI and PSII to moderate fluctuating light, intact leaves were dark-adapted for 30 min and then were exposed to fluctuating light (low light at 100 μmol photons m^-2^ s^-1^ for 2 min and high light at 1294 μmol photons m^-2^ s^-1^ for 2 min) for 60 min. PSI and PSII parameters were measured every 1 min during this period. Before and after photoinhibitory treatments, the PSI activity (*P*_m_) and the PSII activity (*F*_v_/*F*_m_) were measured after 30 min dark adaptation.

To further determine the effects of intense fluctuating light on PSI and PSII activities, values for *P*_m_ and *F*_v_/*F*_m_ were measured after dark adaptation for 30 min. Subsequently, intact leaves were exposed to fluctuating light alternating between 2258 and 94 μmol photons m^-2^ s^-1^ every 5 min for 100 min or constant high light (2258 μmol photons m^-2^ s^-1^) for 100 min. Afterward, leaves were dark-adapted for 30 min and the values of *P*_m_ and *F*_v_/*F*_m_ were measured. Immediately, these leaves were exposed to the above fluctuating light or constant high light for 100 min, and then *P*_m_ and *F*_v_/*F*_m_ were measured after 30 min dark adaptation.

### Statistical Analysis

The results were displayed as mean values of five independent experiments. One-Way ANOVA test was used at α = 0.05 significance level to determine whether significant differences existed between different treatments.

## Results

### Photosynthetic Responses to Moderate Fluctuating Light

**FIGURE 1:**
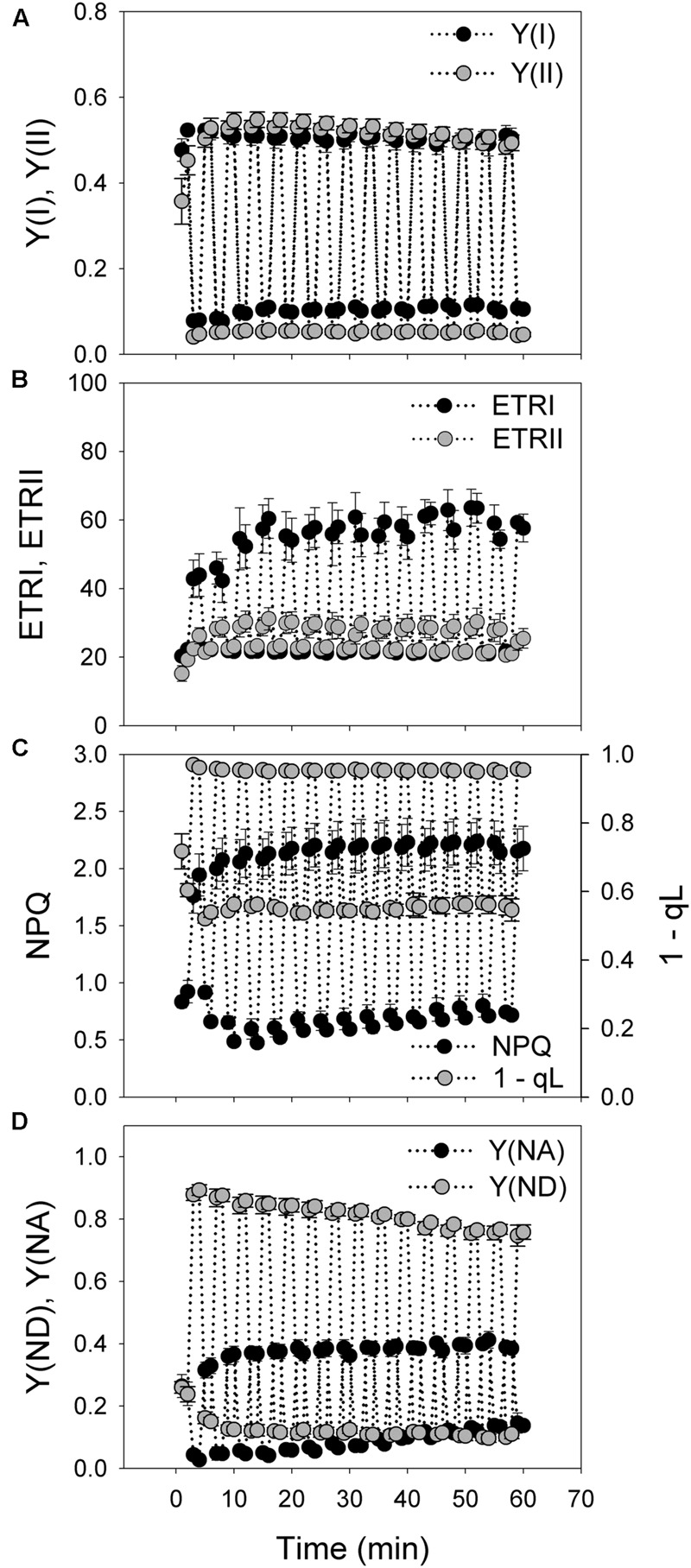
**Responses of photosynthetic parameters to moderate fluctuating light in *Psychotria henryi*. (A)** Y(I) and Y(II); **(B)** ETRI and ETRII; **(C)** NPQ and 1-qL; **(D)** Y(ND) and Y(NA). After dark adaptation for 30 min, leaves were exposed to fluctuating light alternating between 100 and 1294 μmol photons m^-2^ s^-1^ every 2 min for 60 min. Photosynthetic parameters were measured every 1 min. *Y*(I), quantum yield of PSI; *Y*(II), effective quantum yield of PSII; ETRI and ETRII, electron transport rate around PSI (ETRI) and around PSII (ETRII); NPQ, non-photochemical quenching; *Y*(ND), the fraction of P700 that is already oxidized by actinic light; *Y*(NA), the fraction of P700 that cannot be oxidized due to acceptor side limitation. The means ± SE were calculated from at least four plants.

Under fluctuating light alternating between 100 and 1294 μmol photons m^-2^ s^-1^ every 2 min for 60 min, values of *Y*(I), *Y*(II), ETRI, ETRII were constant at 100 μmol photons m^-2^ s^-1^ and gradually increased in the initial 12 min when exposed to 1294 μmol photons m^-2^ s^-1^ (**Figures 1A,B**). NPQ quickly increased from the low levels at 100 μmol photons m^-2^ s^-1^ to the high levels at 1294 μmol photons m^-2^ s^-1^ in 1 min (**Figure 1C**), suggesting the rapid generation of ΔpH. The value of 1 – qL was maintained at high levels at high-light phases and subsequently decreased to low levels at low-light phases (**Figure 1C**), indicating the rapid regulation of the redox state of the plastoquinone pool. Similarly, the P700 redox state under fluctuating light was regulated quickly in response to changes in light conditions. *Y*(ND) fast increased to high levels during the high-light phases and decreased to low levels during the subsequent low-light phases (**Figure 1D**). Similarly, *Y*(NA) rapidly decreased to low levels during the high-light phases (**Figure 1D**).

**FIGURE 2:**
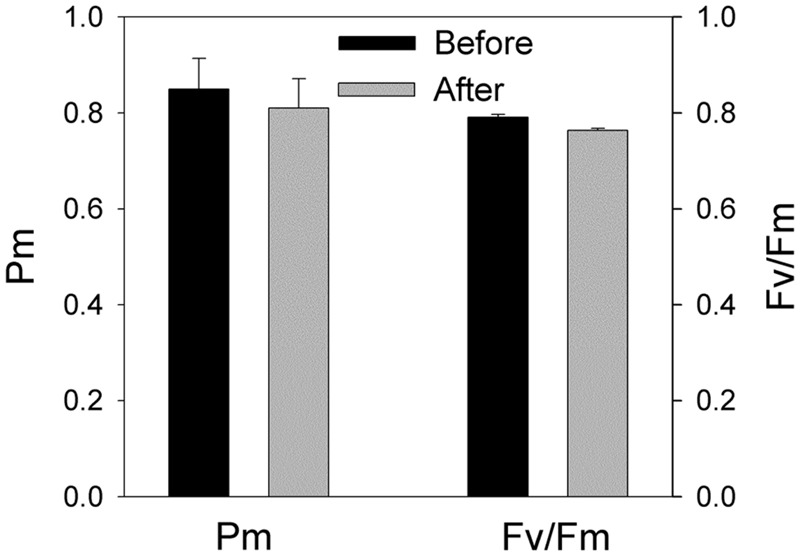
**Effects of moderate fluctuating light on PSI and PSII activities.** The maximum photo-oxidizable P700 (*P*_m_) and the maximum quantum yield of PSII (*F*_v_/*F*_m_) were measured before and after treatment with moderate fluctuating light as shown in **Figure 1**. Values were measured following 30 min dark adaptation. The means ± SE were calculated from at least four plants.

The value of ETRI approximately equaled that of ETRII at the low light of 100 μmol photons m^-2^ s^-1^. However, ETRI was two times higher than ETRII when illuminated at 1294 μmol photons m^-2^ s^-1^ (**Figure 1B**). These results suggested that the shade-establishing species *P. henryi* showed significantly activation of CEF around PSI under high light, which was confirmed by the high levels of *Y*(ND) and low levels of *Y*(NA) at high light. Furthermore, the rapid changes in NPQ, 1 – qL, *Y*(ND), and *Y*(NA) further implied the physiological roles of CEF under fluctuating light. Interestingly, after exposure to this moderate fluctuating light (100 μmol photons m^-2^ s^-1^ for 2 min and 1294 μmol photons m^-2^ s^-1^ for 2 min) for 60 min, both *P*_m_ and *F*_v_/*F*_m_ were remained stable (**Figure 2**), indicating that PSI and PSII activities were insusceptible to short time moderate fluctuating light in the shade-establishing tree species *P. henryi*.

### Photosynthetic Responses to Intense Fluctuating Light

**FIGURE 3:**
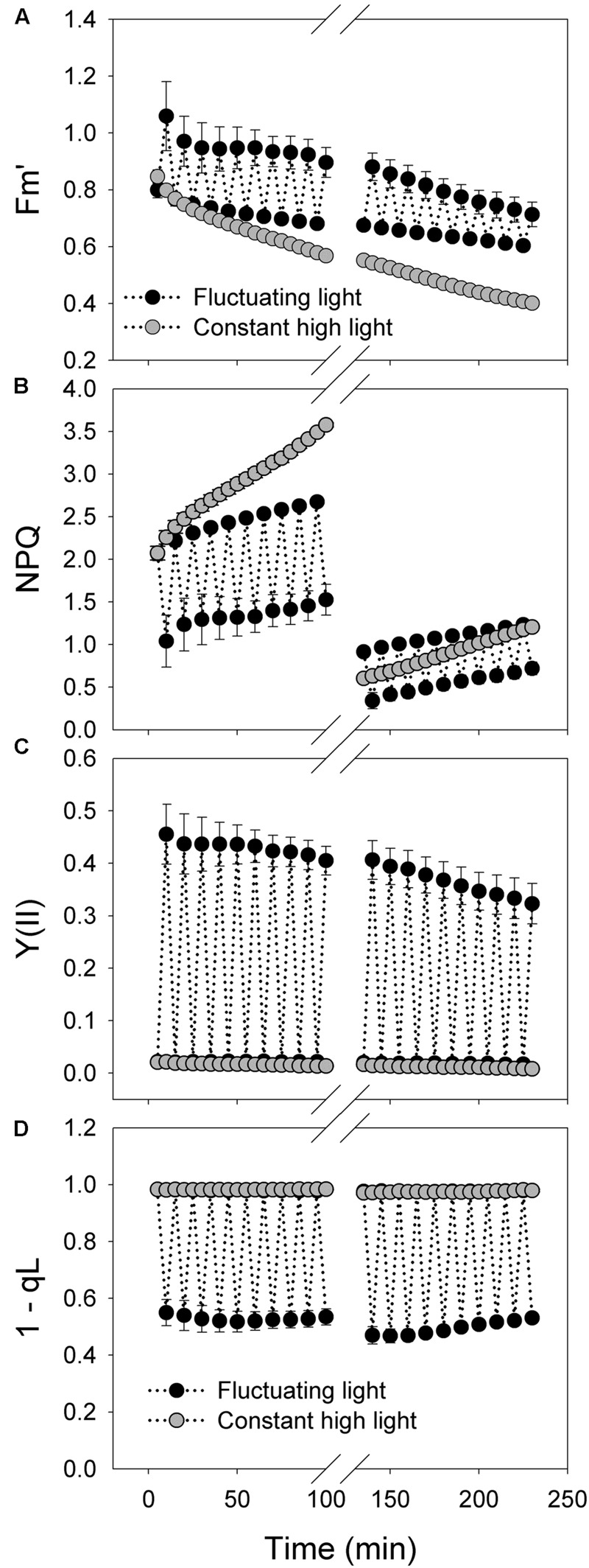
**Response of PSI photosynthetic parameters to intense fluctuating light in *P. henryi*. (A)** Fm’; **(B)** NPQ; **(C)** Y(II); **(D)** 1-qL. After dark adaptation for 30 min, leaves were exposed to fluctuating light alternating between 2258 and 94 μmol photons m^-2^ s^-1^ every 5 min for 100 min. Then, leaves were incubated in darkness for 30 min, and the values of *P*_m_ and *F*_v_/*F*_m_ were measured. Afterward, leaves were treated with fluctuating light alternating between 2258 and 94 μmol photons m^-2^ s^-1^ every 5 min for 100 min. $F_{m}^{\prime }$, maximum fluorescence at actinic light; NPQ, non-photochemical quenching; *Y*(II), effective quantum yield of PSII; 1 – qL, the redox state of plastoquinone pool. The means ± SE were calculated from four plants.

The value of $F_{m}^{\prime }$ decreased at a slower rate during the treatment with fluctuating light alternating between 2258 and 94 μmol photons m^-2^ s^-1^ compared with the treatment with constant light of 2258 μmol photons m^-2^ s^-1^ (**Figure 3A**). Because NPQ is calculated as NPQ = (*F_m_–*$F_{m}^{\prime }$)/$F_{m}^{\prime }$, the larger decrease in $F_{m}^{\prime }$ apparently leads to the faster increase in NPQ under constant high light (**Figure 3B**). However, this increase in NPQ is not truth owing to the decrease in *F*_m_ after high light treatment. After treatment with fluctuating light or constant for 100 min, the actual value of NPQ at 2258 μmol photons m^-2^ s^-1^ largely decreased, as shown by comparison between NPQ values at 5 and 135 min (**Figure 3B**). Under fluctuating light, NPQ can be activated quickly during the high-light phases, suggesting the activation of CEF. During the treatment period of 0–100 min at fluctuating light, the value of *Y*(II) at 94 μmol photons m^-2^ s^-1^ changed little (**Figure 3C**). However, during the treatment period of 100–200 min, the value of *Y*(II) at low-light phases gradually decreased with increasing time (**Figure 3C**). The redox state of the plastoquinone pool (1 – qL) changed slightly during the treatments with constant high light (**Figure 3D**), suggesting the accumulation of reducing power in photosynthetic electron transport system. By comparison, the redox state of the plastoquinone pool was rapidly regulated under fluctuating light, indicating that the accumulated reducing power during the high-light phases can be dissipated during the subsequent low-light phases.

**FIGURE 4:**
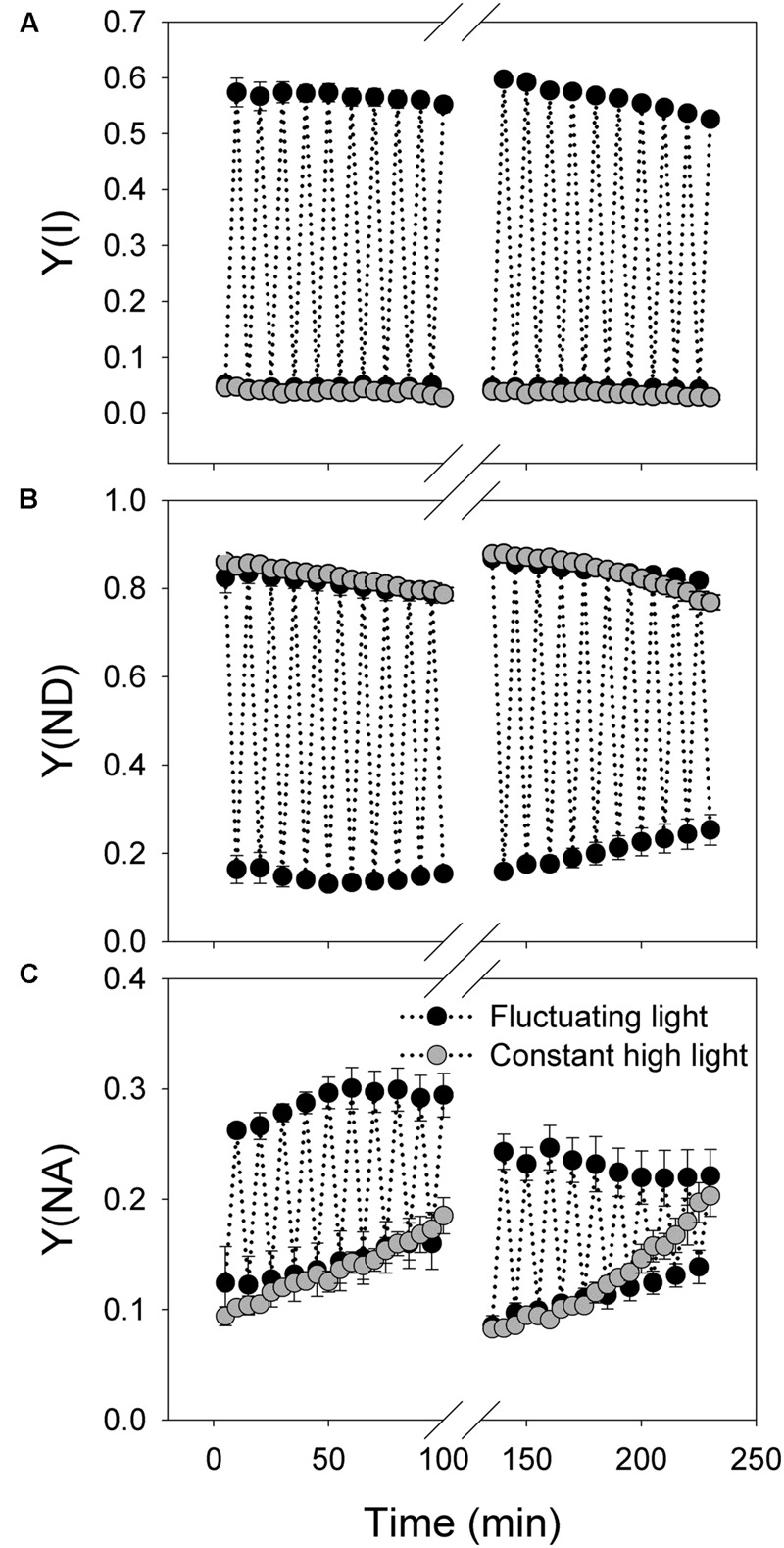
**Response of PSI photosynthetic parameters to intense fluctuating light in *P. henryi*. (A)** Y(I); **(B)** Y(ND); **(C)** Y(NA). After dark adaptation for 30 min, leaves were exposed to fluctuating light alternating between 2258 and 94 μmol photons m^-2^ s^-1^ every 5 min for 100 min. Then, leaves were incubated in darkness for 30 min, and the values of *P*_m_ and *F*_v_/*F*_m_ were measured. Afterward, leaves were treated with fluctuating light alternating between 2258 and 94 μmol photons m^-2^ s^-1^ every 5 min for 100 min. Photosynthetic parameters were measured every 5 min. *Y*(I), quantum yield of PSI; *Y*(ND), the fraction of P700 that is already oxidized by actinic light; *Y*(NA), the fraction of P700 that cannot be oxidized due to acceptor side limitation. The means ± SE were calculated from four plants.

The trend of change in *Y*(I) during treatments with fluctuating light and constant light was similar to *Y*(II) (**Figure 4A**). *Y*(ND) changed regularly according to light condition under fluctuating light and gradually decreased under constant high light treatment (**Figure 4B**). The value of *Y*(NA) at 2258 μmol photons m^-2^ s^-1^ increased gradient with increasing time of treatments under both fluctuating and constant light (**Figure 4C**). Notably, the increase in *Y*(NA) under constant high light was faster than under fluctuating light. Similar to *Y*(ND), *Y*(NA) was regulated regularly under fluctuating light.

**FIGURE 5:**
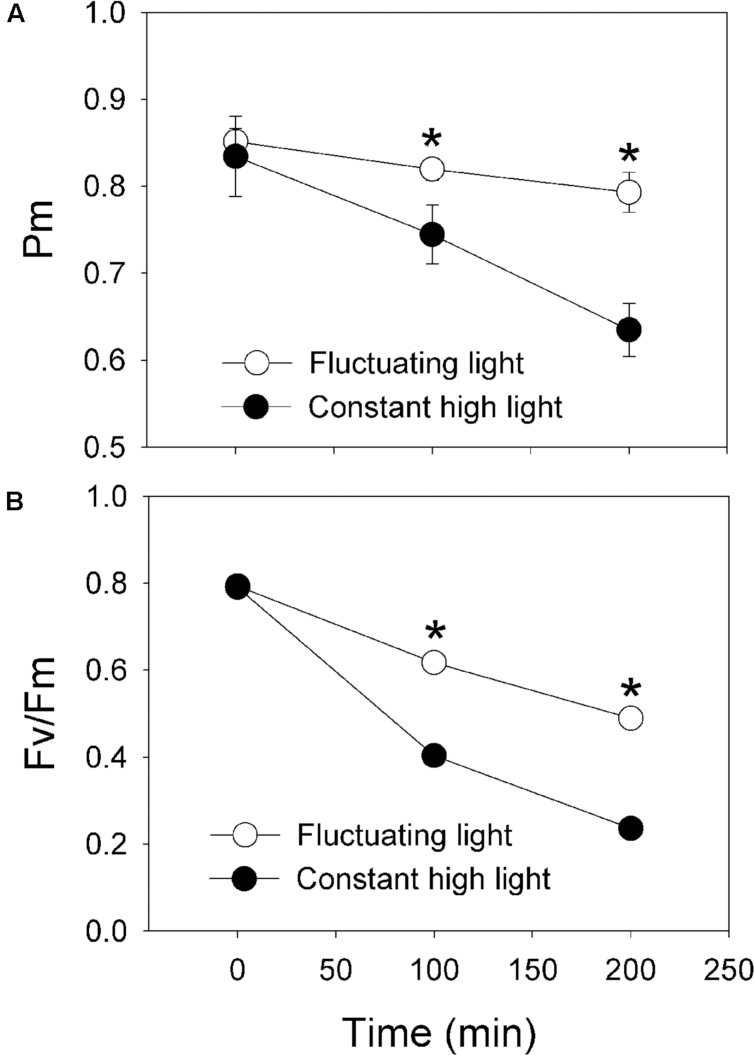
**Effects of intense fluctuating light on PSI **(A)** and PSII **(B)** activities.** The maximum photo-oxidizable P700 (*P*_m_) and the maximum quantum yield of PSII (*F*_v_/*F*_m_) were measured before and after treatment with moderate fluctuating light as shown in **Figures 3** and **4**. Values were measured following 30 min dark adaptation. The means ± SE were calculated from at least four plants.

After exposure to fluctuating light alternating between 2258 and 94 μmol photons m^-2^ s^-1^ every 5 min for 100 and 200 min, the value of *P*_m_ only decreased by 4 and 7%, respectively (**Figure 5A**). Meanwhile, the value of *F*_v_/*F*_m_ decreased to 0.62 and 0.49 (**Figure 5B**). By comparison, after treatment with constant light (2258 μmol photons m^-2^ s^-1^) for 100 and 200 min, *P*_m_ decreased by 11 and 24%, respectively (**Figure 5A**). Concomitantly, *F*_v_/*F*_m_ decreased to 0.40 and 0.24, respectively (**Figure 5B**). These results strongly indicate that (1) PSI is insusceptible to fluctuating light in *P. henryi*; (2) both PSI and PSII are more sensitive to constant high light than fluctuating light in *P. henryi*; (3) photoinhibition of PSI under constant high light cannot be prevented by the activation of CEF.

## Discussion

Photosystem I activity plays important roles in sustaining photoprotection and plant growth (Yamori and Shikanai, 2016). Photoinhibition of PSI activity significantly affects photoprotection, CO_2_ assimilation rate, plant biomass (Brestic et al., 2015; Zivcak et al., 2015; Yamori et al., 2016). Our previous studies indicated that PSI activity is sensitive to high light in a shade-establishing tropical tree species *P. rubra*, suggesting that photoinhibition of PSI is an important mechanism underlying why shade-establishing species cannot survive under high light (Huang et al., 2015b). Here, we examined the photosynthetic responses to fluctuating light in the shade-establishing tropical tree species *P. henryi*, a coexisting relative species of *P. rubra*. Interestingly, we found that PSI and PSII were insusceptible to moderate fluctuating light in *P. henryi* (**Figure 2**). However, constant high light led to significant decrease in PSI activity in *P. henryi* (**Figure 5A**). These results strongly indicate that PSI is a key determiner of photosynthetic acclimation to high light under fluctuating light or constant high light in the shade-establishing species *P. henryi*. Furthermore, photoinhibition of PSI and PSII was significantly stronger after treatment with constant high light than fluctuating light in *P. henryi* (**Figure 5B**), which is different from the phenomenon in model plant *A. thaliana* (Kono et al., 2014) and rice (Yamori et al., 2016). As a result, the underlying mechanism of PSI photoinhibition in *P. henryi* is probably different from *A. thaliana* and rice.

### Response of Photosystem II to Fluctuating Light

We found that PSII activity was insusceptible to moderate fluctuating light in *P. henryi* (**Figure 2**). Photoinhibition of PSII occurs only when the rate of photodamage exceeds the rate of repair. After exposure to moderate fluctuating light (100 and 1294 μmol photons m^-2^ s^-1^ every 2 min) for 60 min, *F*_v_/*F*_m_ only showed a little decrease in *P. henryi* (**Figure 2**), indicating that the photodamage induced by high-light (1294 μmol photons m^-2^ s^-1^) phases can be fast recovered at the subsequent low-light (100 μmol photons m^-2^ s^-1^) phases. Furthermore, this result suggested that exposure to 1294 μmol photons m^-2^ s^-1^ for 2 min hardly caused significant accumulation of ROS, because the repair of photodamaged PSII can be inhibited by ROS (Nishiyama et al., 2001, 2005, 2011). Similarly, PSII photoinhibition was significantly stronger under constant high light than under fluctuating light (**Figure 5B**), indicating that the low-light phases significantly affect the extent of PSII photoinhibition. As indicated in **Figure 1**, the maximum value of ETRII was approximately 30 μmol electrons m^-2^ s^-1^, indicating the relatively low ability to utilize light energy in *P. henryi*. Furthermore, the value of 1 – qL was maintained at high levels when exposed to high light (**Figure 3D**), suggesting that the plastoquinone pool was over-reduced due to accumulation of reducing power in the photosynthetic electron transport system. As a result, PSII in *P. henryi* was very sensitive to constant high light (**Figure 5B**), owing to excess production of ROS (Asada, 1999; Murata et al., 2007). Under fluctuating light, the reducing power accumulated during the high-light phases can be dissipated during the subsequent low-light phases, as indicated by the fast decrease of 1 – qL (**Figures 1C,D**). As a result, the low-light phases favor the scavenging of ROS and the fast repair of PSII. Additionally, the rate of PSII photodamage is dependent on light intensity (Allakhverdiev and Murata, 2004), and thus decreased at low-light phases. Under constant high light, the total incident light energy received by leaves is much higher than under fluctuating light, leading to stronger PSII photodamage and generation of ROS. Consequently, we assumed that the stronger PSII photoinhibition under constant high light was caused by the higher rate of PSII photodamage and the ROS-caused inhibition of PSII repair. By comparison, the low-light phases under fluctuating light diminished the rate of PSII photodamage and favored the repair of photodamaged PSII.

### Response of Photosystem I to Fluctuating Light

At present, it is thought that cyclic electron flow around PSI (CEF) is essential for photosynthesis in angiosperm plants (Munekage et al., 2002, 2004; Suorsa et al., 2012; Tikkanen and Aro, 2014; Yamori and Shikanai, 2016). As indicated in *A. thaliana*, the redox state of the NADPH pool regulates PGR5-dependent CEF (Bendall and Manasse, 1995; Johnson, 2005; Okegawa et al., 2008). Under conditions in which the ration of NADP^+^/NADPH ratio decreases, PGR5-dependent CEF is activated to help the generation of ΔpH (Okegawa et al., 2008), which favors ATP synthesis and photoprotection (Munekage et al., 2002; Takahashi et al., 2009; Yamori et al., 2011; Nishikawa et al., 2012; Walker et al., 2014; Huang et al., 2015a, 2016a,b). Impairment of PGR5-dependent CEF induces mortality of *A. thaliana* not only under high light (Munekage et al., 2002), but also under fluctuating light (Suorsa et al., 2012). Therefore, based on these previous studies, CEF has been regarded as an essential mechanism sustaining photosynthesis and plant growth under conditions of high light and fluctuating light (Yamori and Shikanai, 2016). On the other hand, it has been believed that wild-type plants are capable of protecting photosystem I (PSI) under high light or fluctuating light. In the studies of *P. henryi* species, CEF was significantly activated under high light, as indicated by the values of ETRI, ETRII, *Y*(ND), and *Y*(NA) (**Figure 1**). However, it also showed significantly PSI photoinhibition after treatment with constant high light (**Figure 5A**). Furthermore, recent studies found that PSI photoinhibition also occurred in wild-type of *A. thaliana* (Kono et al., 2014) and rice (Kono et al., 2014) after exposure to fluctuating light, and in a shade-establishing species *P. rubra* (Huang et al., 2016d). These results indicated that CEF cannot completely prevent PSI photoinhibition in some wild-type plants.

We found that, PSI was sensitive to constant high light in the shade-establishing tropical tree species *P. henryi* (**Figure 5A**), whereas short-term fluctuating light hardly inactivated PSI activity (**Figures 2** and **5A**). As a result, PSI is a key determiner of photosynthetic responses to fluctuating light and constant high light in *P. henryi*. These results are largely opposite to the phenomenon in the *pgr5* plants of both *A. thaliana* and rice, in which PSI is more susceptible to fluctuating light than to constant high light (Kono et al., 2014; Yamori et al., 2016). The high-light induced PSI photoinhibition in *pgr5* plants occurs mainly due to over-reduction of P700 reaction centers (Munekage et al., 2002, 2004). Recently, another mechanism of PSI photoinhibition in *pgr5* and *crr6* plants has been proposed. In WT plants, CEF-dependent generation of ΔpH induces acidification of thylakoid lumen, as a result, photosynthetic electron flow from PSII to PSI via Cyt *b_6_*/*f* is depressed owing to “photosynthetic control” (Suorsa et al., 2012; Tikkanen and Aro, 2014; Yamori et al., 2016). By comparison, our recent studies found that PSI photoinhibition is more related to the electron flow from PSII to PSI rather that P700 redox state in the shade-establishing tropical tree species *P. rubra* (Huang et al., 2015b, 2016d). Therefore, the main mechanism of PSI photoinhibition largely differs between *P. rubra* and *A. thaliana*. Interestingly, the response of PSI to fluctuating light and constant high light in the studied *P. henryi* species also differs from that of *A. thaliana*, implying the different mechanism of PSI photoinhibition between *P. henryi* and *A. thaliana*.

Under high light, P700 redox state plays an important role in determining the sensitivity of PSI to photoinhibition in model plant *A. thaliana* (Munekage et al., 2002; Suorsa et al., 2012). In *pgr5* and *crr6* plants of rice, the PSI reaction centers remained fully reduced during the high-light phases, whereas in the WT plants, the PSI reaction centers became normally oxidized when transited to high light (Yamori et al., 2016). In the present studied *P. henryi* species, the occurrence of PSI photoinhibition under constant high light was accompanied with high levels of P700 oxidation ratio (**Figures 4B** and **5A**). As a result, unlike *pgr5* plants of *A. thaliana*, PSI photoinhibition is less related to P700 oxidation ratio in *P. henryi*. This result is consistent with our recent studies on a coexisting relative *P. rubra* species, in which PSI photoinhibition under high light is mainly determined by the electron flow from PSII to PSI (Huang et al., 2016d). Under fluctuating light, the values of 1 – qL decreased quickly to low levels during the low-light phases (**Figures 1C** and **3D**), indicating that the reducing power that accumulates during high-light phases can be dissipated quickly during the subsequent low-light phases. As a result, the ROS production was diminished during the low-light phases, and then over-accumulation of ROS was avoided in *P. henryi*, which was confirmed by the stability of PSI and PSII after treatment with moderate fluctuating light for 60 min (**Figure 2**). Under constant high light, the total incident light energy that leaves receive is much higher than that receive under fluctuating light. And the over-reduction of photosynthetic electron chains, as indicated by high levels of 1 – qL, induced the production and accumulation of ROS and then aggravated PSII photoinhibition (**Figure 5B**). Based on the results of stronger PSI and PSII photoinhibition under constant high light than under fluctuating light (**Figure 5A**), we propose that PSI photoinhibition in *P. henryi* is largely dependent on the accumulation of ROS. In other words, the two coexisting relative shade-establishing species *P. henryi* and *P. rubra* probably have the same mechanism of PSI photoinhibition under high light.

As discussed above, ROS are responsible for the photoinhibition of PSI in *P. henryi*. The significant PSI photoinhibition under high light indicated that ROS generated under high light could not be scavenged quickly by anti-oxidative enzymes, leading to accumulation of ROS. This implies the insufficient capacity of the anti-oxidative system in the chloroplast in shade-establishing plants. As a result, future studies are still needed to examine the relationship between high-light-induced PSI photoinhibition and the capacity of anti-oxidative system in shade-establishing species. Although PSI photoinhibition is inevitable in the shade-establishing species *P. henryi* and *P. rubra*, PSI photoinhibition also represent some kind of protective mechanisms against long lasting over-reduction of PSI acceptor side, diminishing production of enormous quantity of ROS and preventing extensive damage of cell structures and cell death in stress conditions (Brestic et al., 2016). As a result, PSI photoinhibition may be a protective response in shade-establishing species when exposed to short-term constant high light stress.

### Physiological Roles of CEF in Shade-Establishing Species

In the model plant *A. thaliana*, the physiological functions of CEF under constant high light and fluctuating light have been clarified (Munekage et al., 2002, 2004; Suorsa et al., 2012; Kono et al., 2014; Tikkanen et al., 2014; Yamori and Shikanai, 2016). CEF plays a central role in the regulation of linear electron flow via the down-regulation of the Cyt *b*_6_/*f* complex upon generation of ΔpH. In *pgr5*-plants of *A. thaliana*, treatment with constant high light for several hours leads to severe photodamage of PSI (Munekage et al., 2002; Tikkanen et al., 2014) and PSII (Takahashi et al., 2009). Moreover, PSI is very susceptible to fluctuating light in *pgr5*-plants of both *A. thaliana* and rice (Suorsa et al., 2012; Kono et al., 2014; Yamori et al., 2016). In two light-demanding tropical tree species *Erythrophleum guineense* and *Khaya ivorensis*, CEF was flexibly modulated during daytime under fluctuating light conditions and then PSI activity was maintained stable during the daytime (Huang et al., 2016a). Here, in the shade-establishing *P. henryi* species, the activation of CEF during high-light phases induced the rapid formation of ΔpH (i.e., NPQ) across the thylakoid membranes within 1 min (**Figure 1**), prevents PSI and PSII photoinhibition from moderate fluctuating light (100 and 1294 μmol photons m^-2^ s^-1^ every 2 min for 60 min). These results suggest that CEF is critical for photosynthetic acclimation to fluctuating light in both light-demanding and shade-establishing plants. Under constant high light, PSI photoinhibition can be avoided owing to the activation of CEF in light-demanding plants. However, CEF cannot completely prevent PSI photoinhibition in shade-establishing species such as *P. henryi* (**Figure 5A**) and *P. rubra* (Huang et al., 2015b, 2016d).

## Conclusion

In the present study, we examined the responses of PSI and PSII to fluctuating light and constant high light in the shade-establishing tropical tree species *P. henryi*. The stability of PSI and PSII activities under moderate fluctuating light (**Figures 1** and **2**) strongly indicated that shade-establishing species have the ability to avoid photoinhibition during the period of short-term light spot. The significantly stronger photoinhibition of PSI and PSII under constant high light indicated that the mechanisms of PSI photoinhibition in *P. henryi* are different from *A. thaliana* and rice. In *P. henryi*, PSI photoinhibition is more related to the accumulation of ROS rather than to P700 redox state, which is similar to its relative *P. rubra* species (Huang et al., 2016d). Furthermore, our results confirm the important roles of CEF in photosynthetic acclimation to fluctuating light.

## Author Contributions

WH and S-BZ conceived and designed research. WH and Y-JY conducted experiments. WH and Y-JY analyzed data. WH, HH, and S-BZ wrote the manuscript.

## Conflict of Interest Statement

The authors declare that the research was conducted in the absence of any commercial or financial relationships that could be construed as a potential conflict of interest.

The reviewer MR and handling Editor declared their shared affiliation, and the handling Editor states that the process nevertheless met the standards of a fair and objective review.
